# Mixing and Packing Amalgam*Note—This extract is taken from a paper read before the Wisconsin State Dental Society, and is printed in full in the November 1914, Dental Review.

**Published:** 1914-12-15

**Authors:** W. E. Harper


### MIXING AND PACKING AMALGAM*

BY W. E. HARPER. D.D.S.

Proper mixing is best done by the use of a deep glass mortar, the inner surface of which has been slightly dulled (not ground) and pestle of such design as to afford a firm

---

*Note—This extract is taken from a paper read before the Wisconsin State Dental Society, and is printed in full in the November 1914, *Dental Review.*

grasp being taken of its handle. The head of the pestle should also be slightly dulled. The time required, and the rapidity of movement of the pestle necessary for thorough amalgamation, make the use of the shallow mortar impracticable because of the danger of loss of some of the contents during the operation. A rough inner surface of the mortar tends to grind the alloy, which is objectionable to say nothing of the extreme difficulty in completely removing the plastic mass, and keeping the mortar clean.

Mixing in the deep glass mortar should be done thoroughly by a rapid movement of the pestle in such manner as to keep the mix always at the bottom of the mortar, this will necessitate the constant rubbing or shaking down of that portion which collects on the sides. The force exerted in mixing should not be one of grinding, but of moderate rubbing together, to accomplish the most complete amalgamation. Such mixing should be continued for two minutes by the watch as a minimum length of time, followed by kneading the mass in the hand one minute, this time requirement applies to all mixes made of from ten to thirty grains of alloy, but a mix of less than ten grains might require a little less time, and a mix of more than thirty grains will require more time.

If the proper proportion of alloy and mercury have been used and the necessary time and thoroughness have been taken in the amalgamation, the mass will show a degree of plasticity indicated by slight sloppiness, a condition of plasticity most favorable to uniform results in adaptation to cavity walls.

The mass should not, however, be so plastic as not to retain its form when made into a roll, as such a condition is considered excessively sloppy. If, in the final kneading any crepitus or indication of setting is felt, the mix is unfavorable to adaptation except for very small cavities.

Uniformity of results in adaptation to cavity walls appears to be more dependent upon the condition of plasticity during the packing than has been recognized. These observations are the result of extensive experiments with the various well known alloys, and it is not surprising that they have been conclusive to those operators who have participated in the work.

My records show that at least ninety per cent of the operators whose work has come under my obersvation, make an incomplete mix, due in almost every instance to insufficient time being taken, and to the limitation of rapid rubbing which the use of a shallow mortar or only a hand mix affords.

This incomplete amalgamation is probably the cause of those bulk changes which time develops in some of our fillings, and this faulty technic may also explain many discrepancies in reported tests recorded on the micrometer which do so many of our reliable manufacturers a great injustice.

Packing.—The packing should be done with flat serrated faced pluggers, only a very limited number being necessary to meet the full requirements of an every day practice. The following selection is suggested as sufficiently complete, viz: A large plugger 3 to 4 millimeters in diameter.

A mediam sized plugger, 2.5 millimeters in diameter.

A small plugger of the approximate size of 12x18 tenths of a millimeter in diameter, made in some angular form to reach well into the angles of a cavity. Such measurements are of course approximate and may be varied to suit the desires of the individual operator.

The character of the plasticity of the amalgam at the start and during the packing is an important factor, and is absolutely essential to gain reasonably uniform results in adaptation. This condition of plasticity with compression appear to be the vital requirements in our procedure, without which I have at all times failed to secure uniform results in all of my experimental work during the past four years. This has also been the experience of all of those who have assisted me in making the tests.

To continue with the packing, take a piece of the very plastic amalgam sufficiently large to cover the floor of the cavity and pack with all the force of the pen grasp, using as large a plugger as possible, taking short steps in concentric order, working from the center of the filling to and around the walls. Remove the excess mercury as it appears upon the surface, repeating the operation as each piece of amalgam is added until the filling is completed. The excess removed during the packing may be used for the finishing of the filling by pinching out the excess mercury. In cavities of irregular outline form, the small angular pluggers must be used to reach well into the angles or irregularities and in so doing great care should be taken to adopt some order of stepping that will insure the use of the small plugger on every part of the surface not reached or condensed by the use of the larger plugger.

The filling should be finished with an excess and allowed a few minutes to set, after which the filling may be trimmed to form, to be polished at a subsequent sitting.

Three details of technique appear to be equally important and essential to secure uniformly perfect and stable adaptation, namely:

First—Thorough mixing, for greater stability, and a decided plasticity of the mix during the packing to make possible reasonably uniform results.

Second—Compression made possible only by the use of the flat faced serrated pluggers, and the use of the matrix if any of the surrounding cavity walls are missing.

Third—An order in the stepping of the plugger which will insure covering the entire surface of the filling, with special attention paid in packing against the walls and in all of the angles of the cavity.

The general packing in proximo-occlusal cavities must necessarily be done with a small plugger, and in such irregular cavities it is very difficult to carry out an order in the stepping of the plugger point that will insure complete condensation. The use of a small plugger is not as favorable to compression of the mass as is the use of a large plugger, because the condensing area of the small pluggers is so reduced as to fail to grasp the amalgam with the result that it crawls from under the condensing force and the mass is more or less chopped up. Enough work with the small plugger will eliminate the naked eye defects, but fails to compress the amalgum into solid adaptation. To overcome these technical difficulties inspires to a continuance of the work.

